# Integrated Molecular Characterization of Gastrointestinal Stromal Tumors (GIST) Harboring the Rare D842V Mutation in PDGFRA Gene

**DOI:** 10.3390/ijms19030732

**Published:** 2018-03-04

**Authors:** Valentina Indio, Annalisa Astolfi, Giuseppe Tarantino, Milena Urbini, Janice Patterson, Margherita Nannini, Maristella Saponara, Lidia Gatto, Donatella Santini, Italo F. do Valle, Gastone Castellani, Daniel Remondini, Michelangelo Fiorentino, Margaret von Mehren, Giovanni Brandi, Guido Biasco, Michael C. Heinrich, Maria Abbondanza Pantaleo

**Affiliations:** 1 “Giorgio Prodi” Cancer Research Center, University of Bologna, Bologna 40138 Italy; valentina.indio2@unibo.it (V.I.); giuseppe.tarantino6@unibo.it (G.T.); milena.urbini2@unibo.it (M.U.); guido.biasco@unibo.it (G.B.); maria.pantaleo@unibo.it (M.A.P.); 2 Division of Hematology and Oncology, Portland VA Health Care System and OHSU Knight Cancer Institute, Oregon Health and Science University, Portland, OR 97239, USA; patterja@ohsu.edu (J.P.); heinrich@ohsu.edu (M.C.H.); 3 Department of Specialized, Experimental and Diagnostic Medicine, Sant’Orsola-Malpighi Hospital, University of Bologna, Bologna 40138, Italy; margherita.nannini@unibo.it (M.N.); maristella.saponara@unibo.it (M.S.); lidia.gatto83@gmail.com (L.G.); giovanni.brandi@unibo.it (G.B.); 4 Pathology Unit, Sant’Orsola-Malpighi Hospital, University of Bologna, Bologna 40138, Italy; donatella.santini@aosp.bo.it; 5 Department of Physics and Astronomy, L. Galvani Center for Biocomplexity, Biophysics and Systems Biology, University of Bologna, Bologna 40138, Italy; italodovalle@gmail.com (I.F.d.V.); gastone.castellani@unibo.it (G.C.); daniel.remondini@unibo.it (D.R.); 6 Laboratory of Oncological and Transplant Molecular Pathology—Pathology Unit, Sant’Orsola-Malpighi Hospital, University of Bologna, Bologna 40138, Italy; michelangelo.fiorentino@aosp.bo.it; 7 Department of Hematology and Medical Oncology, Fox Chase Cancer Center, Temple University Philadelphia, PA 19111, USA; margaret.vonmehren@fccc.edu

**Keywords:** gastrointestinal stromal tumors, D842V, GIST, KIT, PDGFRA, crenolanib

## Abstract

Gastrointestinal stromal tumors (GIST) carrying the D842V activating mutation in the platelet-derived growth factor receptor alpha (*PDGFRA*) gene are a very rare subgroup of GIST (about 10%) known to be resistant to conventional tyrosine kinase inhibitors (TKIs) and to show an indolent behavior. In this study, we performed an integrated molecular characterization of D842V mutant GIST by whole-transcriptome and whole-exome sequencing coupled with protein–ligand interaction modelling to identify the molecular signature and any additional recurrent genomic event related to their clinical course. We found a very specific gene expression profile of D842V mutant tumors showing the activation of G-protein-coupled receptor (GPCR) signaling and a relative downregulation of cell cycle processes. Beyond D842V, no recurrently mutated genes were found in our cohort. Nevertheless, many private, clinically relevant alterations were found in each tumor (*TP53, IDH1, FBXW7, SDH*-complex). Molecular modeling of PDGFRA D842V suggests that the mutant protein binds imatinib with lower affinity with respect to wild-type structure, showing higher stability during the interaction with other type I TKIs (like crenolanib). D842V mutant GIST do not show any actionable recurrent molecular events of therapeutic significance, therefore this study supports the rationale of novel TKIs development that are currently being evaluated in clinical studies for the treatment of D842V mutant GIST.

## 1. Introduction

Approximately 80% of gastrointestinal stromal tumors (GIST) carry pathogenic activating mutations of proto-Oncogene c-Kit (*KIT*), while 5% to 10% harbor activating mutations of the platelet-derived growth factor receptor alpha (*PDGFRA*), both members of the type III class of tyrosine kinase receptors [1,2,3]. The use of KIT/PDGFRA tyrosine kinase inhibitors (TKIs; imatinib, sunitinib, and others) has revolutionized the medical treatment of GIST patients. It is known that the sensitivity to TKIs is strictly correlated with the various types of *KIT/PDGFRA* mutations, with the longest progression-free and overall survival associated to patients whose GIST harbors exon 11 mutations [4,5,6,7,8,9]. Among PDGFRA mutant GIST, different mutations have been described with a variable spectrum of sensitivity to TKIs. Some *PDGFRA* mutations (e.g., V561D or deletion DIMH842-845) are associated to a clear sensitivity to imatinib in vitro and in clinical studies, whereas other alterations confer treatment resistance in vitro (e.g., *PDGFRA* D842V, *PDGFRA* D842Y, or *PDGFRA* DI842-843IM) [4,6,8,10,11,12]. The most common *PDGFRA* mutation is the exon 18 D842V, which is fully resistant to imatinib and sunitinib [9,10]. Patients with metastatic/advanced D842V mutant GIST do not benefit from standard TKIs therapy with a median progression-free survival of 2.8 months with imatinib and a median overall survival of 12.7 months [13]. Recently, a promising anti-proliferative activity of crenolanib, an orally available selective and potent inhibitor of PDGFRA and PDGFRB, was reported [14]. In vitro studies showed that crenolanib was at least 100-fold more potent than imatinib on PDGFRA D842V kinase and other imatinib-sensitive and imatinib-resistant PDGFRA mutant kinases (D842I, D842V, D842Y, DI842-843IM, and deletion I843). Moreover, crenolanib interferes with the KIT signaling loop by down-regulation of ETV1 in GIST cells lines [15]. In phase I clinical studies on advanced solid tumors, crenolanib showed a good tolerability [16,17]. Therefore, crenolanib could be useful for the treatment of patients with GIST, and in particular those with PDGFRA mutant GIST. Clinical studies of crenolanib are now ongoing (Randomized Trial of Crenolanib in Subjects with D842V Mutated GIST—ClinicalTrials.gov Identifier: NCT02847429). However, currently there are no effective, approved treatments available for patients with PDGFRA D842V mutant GIST. The goal of our work was to examine the molecular background of primary and metastatic D842V mutant GIST using whole transcriptome (RNA-seq) and whole exome sequencing (WES) analysis, in order to describe the molecular signature and to pinpoint any additional genomic event potentially relevant for the treatment of these patients.

## 2. Results

The molecular characterization of D842V mutant GIST was performed by exploring both RNA-seq and WES together. The study involved a total number of 19 tumor samples, obtained from 14 unique patients with PDGFRA D842V mutant GIST, all analyzed by WES. A subgroup of five out of the 19 tumors were also investigated with whole transcriptome sequencing (Table 1).

In particular, we included eight primary tumors (among which are the five tumors analyzed by RNA-seq), 10 metastases, and one tumor of unknown source. All the primary GIST were naive to any treatment, while the metastases were taken from patients previously treated with one or with combinations of Tyrosine Kinase Inhibitors (TKIs) before surgery (only one of the metastases was also treated with chemotherapy).

### 2.1. Gene Expression Profile

**Figure 1:**
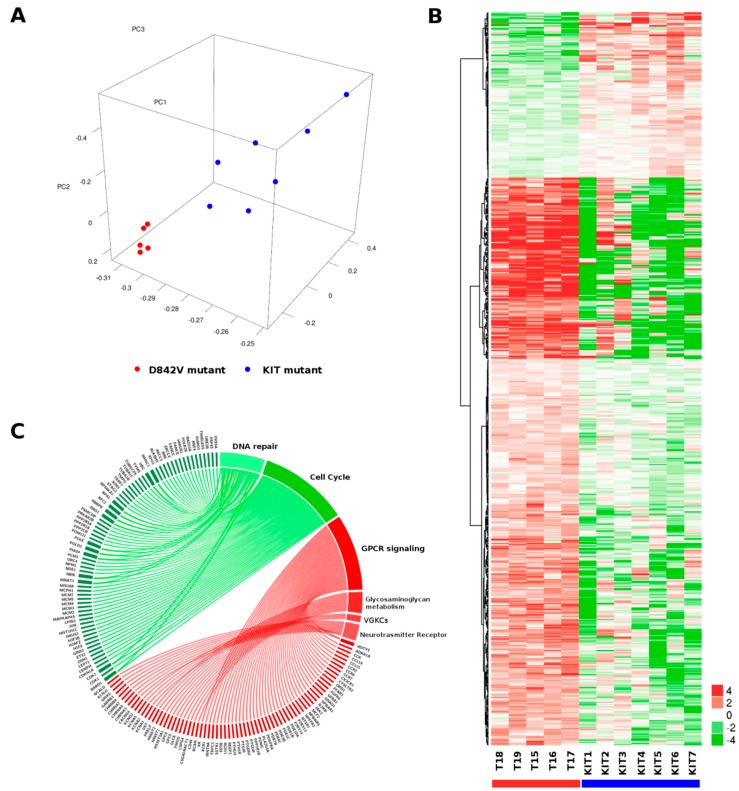
Transcriptome profile of D842V mutant versus KIT mutant GIST. (**A**) Principal component analysis performed on the whole set of expressed genes (*n* = 15,134) shows a very uniform profile of D842V mutant tumors (red dots) that is distinctly separated from KIT mutant GIST (blue dots); (**B**) Heatmap of 494 overexpressed and 144 downregulated genes in D842V GIST; hierarchical clustering was performed to groups genes adopting Manhattan distance and Ward method; (**C**) Graphical representation of genes and pathways emerging as enriched (red) or depleted (green) in D842V mutant GIST. Genes and the corresponding pathways are also reported in Supplementary Table S2.

Gene expression profiling (GEP) was performed by whole transcriptome sequencing to identify specific molecular signatures of five GIST carrying *PDGFRA* D842V mutation (T15–19 corresponding to five unique patients P10–14). To this end, D842V mutant RNA-seq data were compared to a set of seven KIT mutant GIST. The principal component analysis showed a good overlap of the D842V mutant tumors, which is a hallmark of a very homogeneous molecular profile. This is less evident for the KIT mutant tumors, nevertheless we were able to appreciate a discrete spatial separation between the two molecular groups of GIST (Figure 1A), suggesting the presence of two distinct gene expression profiles. Differential expression (DE) analysis identified 494 overexpressed and 144 downregulated genes in the D842V mutant with respect to the KIT mutant GIST (*p*-value < 0.01) (Figure 1B). In order to characterize the gene signature of up/downregulated genes, pathway enrichment analysis was performed using Reactome database (https://reactome.org/)as the database resource (Figure 1C). Table 2 lists the top 10 significantly enriched and depleted pathways (full results are shown in Supplementary Table S1).

We found that the pathways of G-protein-coupled receptor (GPCR) signaling, Glycosaminoglycan metabolism, Voltage-gated potassium channels (VGKCs), and Neurotransmitter Receptor Binding are significantly enriched in the D842V mutant group, while the cell cycle and DNA repair gene sets are enriched in the KIT mutant group. The GPCR signaling pathway includes the majority of leading edge genes. Among them, the most overexpressed genes in the D842V mutant are: Dopamine receptor D1 (*DRD1*, log_2_Ratio = 6.8, *p*-value = 0.00069); Somatostatine receptor 1 (log_2_Ratio = 6.7, *p*-value = 0.00035); Neuropeptides B and W receptor 1 (*NPBWR*, log_2_Ratio = 6.6; *p*-value = 0.0066); and Cholecystokinin (*CCK*, log_2_Ratio = 5.7; *p*-value = 0.0021). We also highlighted quite a large set of chemokines (*CCL19, CCL21, CCR2, CCR6, CCR7, CX3CR1*) and prostaglandins (*PTGDR, PTGER4, PTGIR*) that contribute to the enrichment of GPCR signaling pathway. On the contrary, the cell cycle pathway contains a large number of downregulated genes in the D842V tumors such as the minichromosome maintenance complex genes (MCM family), polymerase genes (*POLE*, *POLD2*) and cyclin-dependent kinase (*CDK1*, *CDK2*, *CDKN1A*).

### 2.2. Fusion Transcript by Whole Transcriptome Sequencing

To further define the transcriptional profile of D842V mutant tumors, we also looked for chimeric transcripts through RNA-seq data. The consensus method highlighted a total of 13 fusion genes in the five tumors analyzed (Supplementary Table S3). We found two recurrent chimeras (*POLA2-CDC42EP* and *CTSC-RAB38*) that did not support any chromosomal rearrangement since they derive from non-pathological read-through transcription. Notably, the large majority of the detected fusion genes (more than 90%) correspond to read-through or conjoined genes that are probably not disease-related. Only one intra-chromosomal rearrangement on chromosome 14 in the tumor T17 was detected (one of the tumors in which chromosome 14 deletion were identified by WES). In particular, two different gene fusion detection algorithm predicted a breakpoint sequence between the 5′UTR of *C14orf159* and the exon 2 of *MGDA2* (Ensembl v91: ENSE00003707948), either leading to the loss of start codon of *MGDA2* in the isoforms carrying that exon as a coding component or allowing the *MGDA2* expression under the control of the *C14orf159* promoter (for the other isoforms). However, the low depth of coverage at breakpoint level and the very poor expression of *MGDA2* suggest that the gene fusion is not functionally relevant.

From a global point of view, the very low rate of chromosomal aberrations leading to chimeric transcripts suggests that D842V tumors have a rather stable genome.

### 2.3. Whole Exome Sequencing

To better investigate the mutational burden of the D842V mutant GIST, we performed the WES analysis on 19 tumor samples and on the matched normal counterpart. Five of the 19 samples were analyzed with the paired-end 100 × 2 approach, while sequencing of the remaining 14 samples was performed with a single-single end strategy at 101bp for a total of 197 Gb (Giga bases), thus leading to an average number of mapped reads per sample of 55 × 10^6^, with an average depth of coverage of 60×.

A total of 316 high-confidence somatic rare variants were found, including coding single nucleotide variants (SNVs), frameshift and non-frameshift insertions and deletions (InDels), and variants at ±3 on the splice sites (average = 17, min–max = 3–26) (Supplementary Table S4).

**Figure 2:**
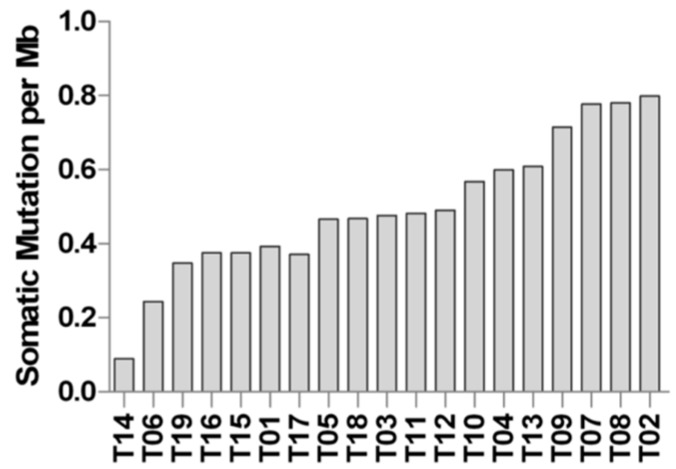
Mutational burden of D842V mutant GIST. The histogram bars indicate the number of somatic mutations per magabases (Mb) of coding region.

Mutational burden was calculated and reported in Figure 2. Most of the samples show a low degree of somatic mutations per Mb of captured coding exome ~0.5, which is comparable to the mutational load of chronic lymphocytic leukemia (CLL), neuroblastoma, and glioblastoma. Moreover, two samples (T06 and T14) showed a very low mutational load, similar to acute lymphocytic leukemia (ALL) and medulloblastoma [18]. However, comparing WES data with RNA-seq, we estimated that only one-third of genes carrying somatic SNVs or InDels were actually expressed in tumor cells.

**Figure 3:**
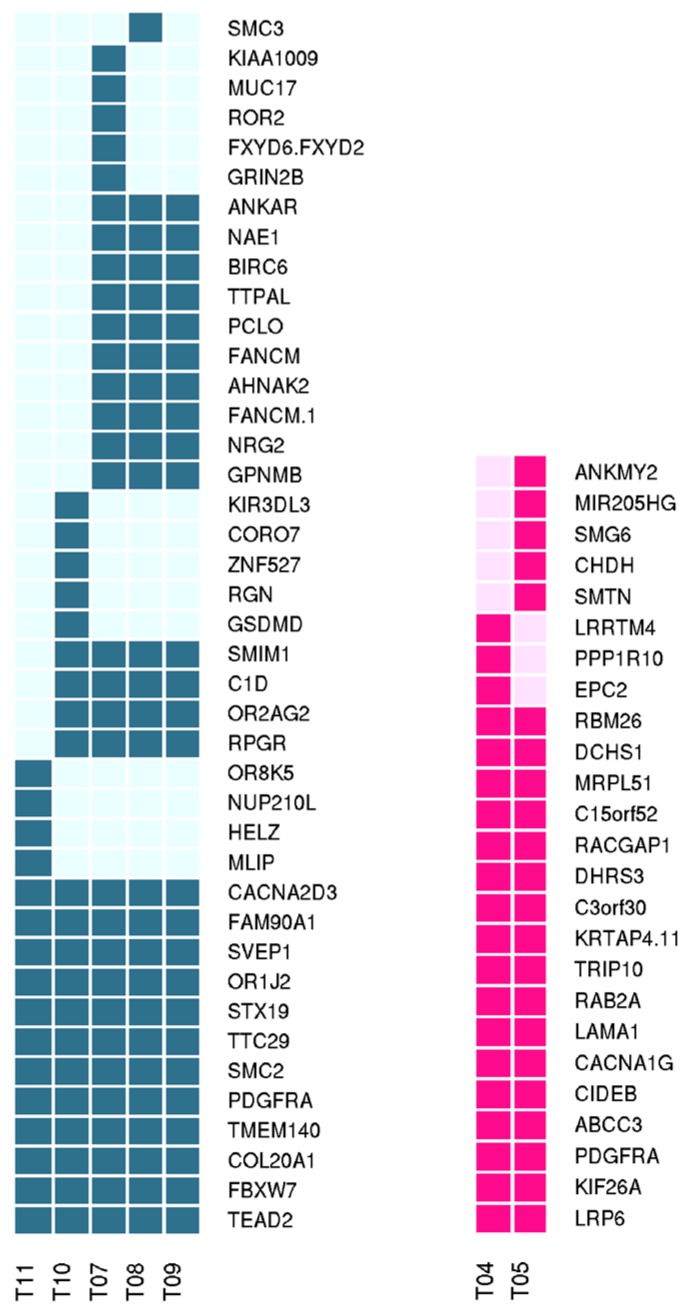
Graphic representation of clonal evolution of metastatic tumors T07–11 and T04–05. The full filled squares indicate the somatic mutations in the corresponding gene (blue and pink respectively for patient P06 and P04).

The only recurrent somatic mutation was located on exon 18 of *PDGFRA*, and corresponded to the expected D842V previously identified by molecular testing procedures. Beyond *PDGFRA* mutations, the results showed three other significantly mutated genes: the transmembrane protein *TMEM140*, the TEA Domain Transcription Factor 2 (*TEAD2*), and the Olfactory Receptor Family 1 Subfamily J Member 2 (*OR1J2*). All the three mutated genes were recurrent in five of the 19 samples that correspond to five geographically distinct metastases of the same patient (samples T07–11 corresponding to patient P06). Although not significantly represented, somatic mutations of *GRIN2B* (Glutamate Receptor, Ionotropic, *N*-Methyl *D*-Aspartate 2B) were found in two different samples (N622D on T06, and V42L on T07). Together with *TMEM140*, *TEAD2*, *OR1J2*, and *GRIN2B*, many other genes were commonly mutated across the tumor samples, however the recurrence always occurred when examining multiple metastatic samples of the same individual (T07–11 or T04–05 corresponding respectively to patients P06 and P04). For these multiple samples of metastasis belonging to same individual, T07–11 (five metastases) and T04–05 (two metastases), the tumor clonal evolution was also evaluated (Figure 3). All the other somatic events were private variants that we can consider as passenger mutations. All the genes with somatic variants were matched with the TARGET database (tumor alterations relevant for genomics-driven therapy—Available onlines: http://archive.broadinstitute.org/cancer/cga/target). By this, we identified three potentially relevant alterations: the Y272C on *IDH1* (T02), the R465H on *FBXW7* (T07–11), and two different mutations on *TP53* (c.993 + 1G > A at exon 10 splice site and a C135G) on sample T13. Moreover, one tumor sample (T06) showed the somatic mutation T60A in the Succinate Dehydrogenase Complex Subunit B (*SDHB*), and interestingly the same patient also carried the R38P germline variant in the subunit D of the same complex (*SDHD*). Both variations on SDH complex genes were previously reported in the archive of ClinVAr (https://www.ncbi.nlm.nih.gov/clinvar/) as germline variants in GIST, hereditary paraganglioma, and pheochromocytoma. In particular, the T60A in *SDHB* was annotated as a single nucleotide variant of uncertain significance, while the R38P in *SDHD* was widely described as pathogenic.

**Figure 4:**
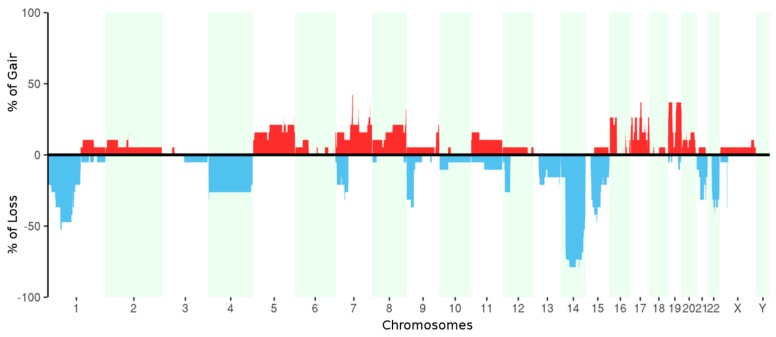
Percentage of samples with copy number gains (red) and losses (blue) for each chromosome.

Copy number alterations were assessed from WES data (Figure 4). We confirmed the commonly known regions of losses in GIST located on chromosome 1, 14, and 22, and we found at least one of these aberrations in ~90% of tumor samples (corresponding to all patients except P05). Moreover, the analysis also showed the loss of chromosome 4, loss of the short arms of chromosomes 7 and 9, and loss of the long arms of chromosomes 15 and 21. In addition, a focal deletion of dystrophin gene (*DMD*) on chromosome X was found in 42% of tumor samples (corresponding to four patients: P03, P06, P07, and P08). This particular aberration is known to be associated with the metastatic tumors [19,20] even if in our series this evidence is not statistically significant due to the small number of samples. We found also numerous copy number gains (in particular on chromosomes 5, 7, 8, 17, and 19) that, interestingly, occurred mostly in metastatic samples.

### 2.4. In Silico Modeling

**Figure 5:**
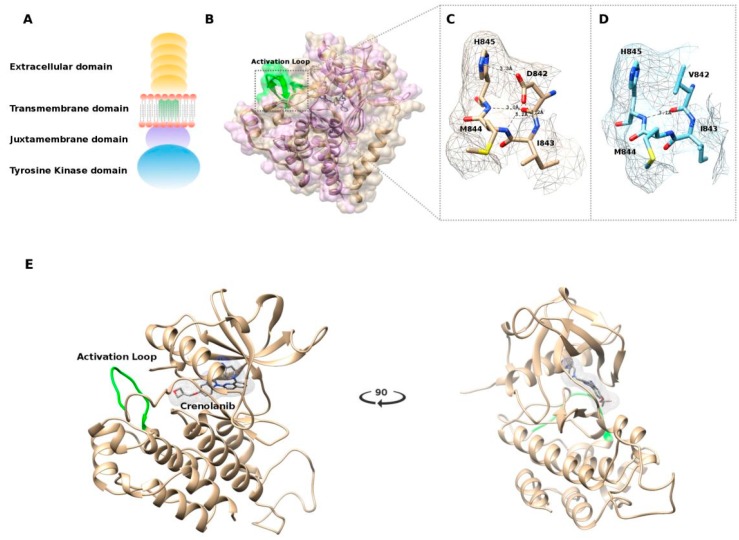
(**A**) Schematic representation of the platelet-derived growth factor receptor alpha (PDGFRA) receptor; (**B**) Structure alignment performed using the jCE algorithm, between the crystallized structure of the c-Kit–Imatinib complex (PDB: 1T46) in purple and the structure of PDGFRA (PDB: 5K5X) in gold. Highlighted in green is the Activation loop; (**C**) Polar interactions of the 842 residue with wild-type Aspartic Acid; (**D**) Polar interactions of the 842 residue carrying the mutated Valine amino acid; (**E**) Representation of the best docked pose of crenolanib in the PDGFRA ATP binding site.

Beyond the whole transcript and whole exome characterization, and due to the absence of other driver molecular events in this GIST subgroup, we investigated the efficacy of the D842V substitution at the protein level, with the aim to define the role of this oncogenic alteration within the peptidic sequence. The single amino acid substitution of aspartic acid with valine at 842 position (D842V) is known to be associated with the resistance to first-line and second-line tyrosine kinase inhibitors (such as imatinib and sunitinib). Since the crystallized structure of the tyrosine kinase domain of PDGFRA (corresponding to the intracellular C-terminal part of the protein, Figure 5A) was recently released (PDB: 5K5X) [21], a protein structure model was created specifically for the D842V mutant and the structure of the complex c-Kit–imatinib (PDB: 1T46) was also aligned in order to ligand imatinib within the PDGFRA structure (Figure 5B).

The mutation D842V is found in the activation loop, known to contain a conserved motif Asp836-Phe837-Gly83 (DFG) that may assume the “in” or “out” conformations corresponding to the active and inactive state of the PDGFRA kinase domain, respectively [21].

X-ray crystallographic studies of protein–ligand interactions demonstrated that imatinib binds to the inactive (DFG-out) conformation of the Type III transmembrane receptor protein tyrosine kinase (RPTK) subfamily including c-Kit and PDGFRA [22]. Our model also predicts that that the mutation D842V leads to the loss of polar interactions involving the residues His845, Ile843, Met844, and Asp846 (Figure 5C,D) that are essential for the stability of the activation loop and to stabilize the DFG-out active conformation. The result is the switch from the DFG-out to the DFG-in conformation activating the kinase, making imatinib incapable of binding to the receptor.

To better understand the molecular basis for the enhanced efficacy of crenolanib versus imatinib against the PDGFRA D842V mutant kinase, we simulated the binding of crenolanib to the modeled PDGFRA in DFG-in conformation. In silico analysis revealed eight different docking clusters of crenolanib at the ATP binding site, and the top scoring docked model was selected (Figure 5E). The docking simulation suggests that crenolanib targets the active conformation of tyrosine kinase subunit of PDGFRA, in which the activation loop is phosphorylated, binding the ATP active residues without involving the adjacent allosteric site that is available only in the inactive conformation (suitable for the type II kinase inhibitors like imatinib and sunitinib) [23].

## 3. Discussion

GIST harboring *PDGFRA* exon 18 D842V are fully resistant to imatinib and sunitinib [9,10]. To date, no approved effective treatments are available for these patients. In addition, no additional molecular events potentially relevant as therapeutic targets are known to be associated with this GIST molecular subtype.

In this study, primary and metastatic D842V mutant GIST were investigated by exploring RNA-seq,WES and molecular modelling adopting a top-down approach. First, whole transcriptome data were analyzed to take a picture of the molecular profile and identify specific signatures of GIST with D842V mutation. Second, each tumor in the D842V mutant cohort was analyzed independently to highlight molecular aberrations such as chromosomal rearrangements (from RNA-seq data), point mutations, and copy number variants (from WES). Lastly, the D842V alteration at the protein level was studied by molecular modelling.

Whole transcriptome analysis showed a very characteristic expression profile of D842V mutant GIST when they are compared with other molecular subgroups of GIST (KIT mutant in our case). In particular, our results highlighted the overexpression of many genes leading to the activation of GPCR signaling. Among these, we noticed genes that suggest a neural differentiation of D842V tumors such as *DRD1*, *SSTR1*, *NPBWR1*, and *CCK*. Moreover, it is known that GIST express somatostatin receptors [24] and that *SSTR1* was widely reported [25] as expressed in the better differentiated gastroenteropancreatic neuroendocrine tumors and associated with a good prognosis. This was suggested also in GIST samples, at least for other somatostatin receptors such as *SSTR2* and *SSTR5*. [24]. This observation, together with a relative downregulation of cell cycle promoting genes, could explain at least in part the clinical evidence of an indolent disease course of D842V mutant GIST with respect other subgroups. The downregulation of cell cycle and DNA repair pathways in the D842V mutant GIST is also important, considering a recent study in which the impairment in these signaling paths was associated with a better response to chemotherapeutic DNA-damaging agents in imatinib-resistant GIST cell lines [26]. Moreover, the upregulation of chemokine and prostaglandin may be a clue of a possible role of tumor microenvironment, and it could suggest the D842V mutant tumor immunogenicity.

Whole exome analysis showed that no actionable molecular events in this population and confirmed that the only recurrent somatic exomic mutation was the D842V. Many other genes variants were found but were determined to be private genetic events.

Notably, additional molecular events such as mutations in *TMEM140* and *TEAD2* were found to be shared in all the five geographically distinct tumor specimens obtained from the same individual. *TMEM140* over-expression was strongly correlated with tumor size, histologic grade, and overall survival time in patients with gliomas [27]. Therefore, *TMEM140* has been proposed as a novel prognostic marker and as a potential therapeutic target for gliomas. *TEAD2* belongs to the protein family that may interact with transcription factors and may play a key role in cancer progression by regulating apoptosis and cell proliferation [28]. Even though the biological functions of *TMEM140* and *TEAD2* seem relevant in cancer development and progression, in D842V mutant GIST these proteins were found mutated in only one patient and therefore no definitive conclusion can be realistically drawn. In addition, *GRIN2B* was mutated in two different samples but this event seems irrelevant in cancer biological processes.

Other relevant events, even though private, emerged by the comparison of the mutated genes with the TARGET archive. In particular, the *IDH1* gene shows a Y272C somatic variant; this gene is known to be frequently mutated in glioma, cholangiocarcinoma, chondrosarcoma, and acute myeloid leukemia, but in those diseases the recurrent mutations is a gain-of-function point mutation at arginine 132. The functional significance of the Y272C mutation is unknown. Conversely, the alteration on *FBXW7* (R465H) is one of the hot-spot mutations previously reported in a large number of different carcinomas and leukemias. However, this alteration is known to confer resistance to specific inhibitors. Finally, we also found biallelic inactivation of tumor suppressor *TP53*, but this mutation is not currently targetable.

Additionally, one tumor sample (T06) harbored a somatic mutation T60A of *SDHB*, and interestingly the same patient carried the germline variant R38P in *SDHD*. *SDHB* and *SDHD* are two subunits of the SDH complex that correspond to the enzyme complex II of the mitochondrial respiratory chain. As is well known, loss of function mutations in these genes are known to characterize the SDH-deficient GISTs lacking *PDGFRA* and *KIT* mutations in the context of Carney Triad or Carney–Stratakis syndrome or sporadic young adult KIT/PDGFRA wild-type GIST, thus mutations in any *SDH* gene are known to be mutually exclusive with *KIT* and *PDGFRA* [29,30]. Recently, one case report showed a patient with D842V mutation that, in addition, harbored two loss of function mutations (germline and somatic) in *SDHB* and the tumor was confirmed as SDH-deficient [31]. Moreover, two case reports are available on patients that harbor mutations in *KIT* receptor in a context of a Carney–Stratakis syndrome in which GIST are supposed to be *KIT/PDGFRA* wild type [32,33]. However, in our case reported here, the SDHB protein expression was not evaluated by immunohistochemistry (IHC) test, so we do not know if these two mutations in *SDHB* and *SDHD* lead to a loss of function of SDH complex.

A percentage of 42% of samples (corresponding to four unique patients) showed a focal deletion of dystrophin gene (on chromosome X). Deletions of dystrophin in KIT/PDGFRA mutant GIST have been previously reported and usually are associated with more advanced clinical stages of disease such as metastatic tumors [19,20]. In this study, the deletion of *DMD* occurred mainly in tumors with a high mitotic index of the primary tumor and in metastatic lesions.

Another relevant point is that none of the analyzed samples, differently from KIT mutant GIST, showed secondary mutations in *PDGFRA*, neither naïve (*n* = 9) nor treated patients (*n* = 5). This observation suggests that the ineffectiveness of drug treatments with type II tyrosine kinase inhibitor is not due to resistance acquisition consequent to the therapy, but indicates this subgroup of GIST are ab initio refractory tumors in which the mechanism of resistance is exclusively related to the primary driver mutation D842V.

Finally, the main finding of this study remains the key role of D842V mutation in this GIST sub population as the main and only relevant event of cancer development. These confirming findings highlight the importance on the development of drugs such as crenolanib that directly inhibit the D842V kinase. By in vitro studies, crenolanib proved to act at least as a 100-fold more potent inhibitor than imatinib on PDGFRA D842V kinase and showed a good tolerability profile in phase I clinical studies on advanced solid tumors [14,16,17]. A phase II study was recently completed to evaluate the antitumor efficacy and pharmacokinetics of crenolanib in patients with D842V mutant GIST, and a phase III trial of crenolanib versus placebo in combination with best supportive care in patients with D842V mutant GIST is now ongoing. To reinforce the proof of principles of these studies, our in silico modeling study revealed eight different docking clusters of crenolanib at the ATP binding site. Crenolanib binds active residues without involving the adjacent allosteric site that is available only in the inactive conformation (suitable for the type II kinase inhibitors like imatinib and sunitinib). This suggests that crenolanib targets the active conformation of the tyrosine kinase subunit of PDGFRA in which the activation loop is phosphorylated. Therefore, patients carrying this mutation should be considered for treatment with a type I tyrosine kinase inhibitor that targets the ATP binding site when the kinase is in the active conformation. In conclusion, in our series of D842V mutant GIST, we did not identify any additional molecular events associated to cancer development that could be considered as potential therapeutic targets. Crenolanib, or other type I tyrosine kinase inhibitors such as BLU-285 [34], should be considered for the treatment of patients with D842V mutant GIST, and further and definitive studies on this approach in clinical settings are expected.

## 4. Materials and Methods

This study was approved by the local institutional ethical committee of Azienda Ospedaliero-Universitaria Policlinico S.Orsola-Malpighi (approval number 113/2008/U/Tess, 30 September 2008 approval code, approval date). All patients provided written informed consent.

Fresh tissues snap-frozen in liquid nitrogen and stored at −80 °C (FF) or formalin-fixed paraffin-embedded (FFPE) specimens of PDGFRA mutant GIST were used for the whole exome analyses. All primary tumors were localized in the stomach, although metastatic tumor samples were analyzed in ~50% of cases. The tumor and patient’s characteristics are reported in Table 1.

Whole Exome Sequencing (WES) was performed on 19 tumor samples previously characterized as harboring *PDFGRA* D842V mutations, and on matched normal counterpart (peripheral blood or stomach). The 19 tumors collected belong to 14 unique patients: 12 patients with one single tumor sample, one patient with two samples (T04 and T05 corresponding to patient P04), one patient with five samples (T07, T08, T09, T10, and T11 corresponding to patient P06). For both patients (P04 and P06), the multiple samples analyzed correspond to different geographically distinct specimens available. Additionally, whole transcriptome analysis was performed on five of the above mentioned samples (T15, T16, T17, T18, T19).

### 4.1. Whole-Transcriptome Paired-End RNA Sequencing and Whole Exome-Sequencing

Total RNA was extracted from tumor specimens with RNeasy Mini Kit (Qiagen, Milan, Italy), then cDNA libraries were synthesized from 250 ng total RNA with TruSeq RNA Sample Prep Kit v2 (Illumina, San Diego, CA, USA) according to the manufacturer’s recommendations. Sequencing by synthesis was performed on HiScanSQ sequencer (Illumina) at 75 bp in paired-end mode. Whole-transcriptome sequencing yielded a total of 32 Giga Bases and an average of 84 millions of short reads with an average depth of coverage of 45×

WES was performed on DNA isolated from fresh frozen and FFPE tumor tissue and from matched normal peripheral blood or stomach DNA. Whole exome libraries were prepared applying different protocols and using two different sequencing platforms: Nextera Rapid Capture Exome Enrichment (Illumina) was adopted on five out of 19 samples that were sequenced on Illumina HiScanSQ at 2 × 100 bp read length; eight out of 19 libraries were prepared with Nimblegen SeqCap v02 (Roche, Pleasanton, CA, USA), and six out of 19 with Nimblegen SeqCap v03 (Roche, Pleasanton, CA, USA) and were run on HiSeq2000 Illumina platform at 100 bp in single-end. For all the three capturing systems, the exome enrichment was performed according to manufacturer’s protocols.

### 4.2. Bioinformatic Analysis

Data analysis was performed on local server CentOS5 Linux, adopting a customized full open source bioinformatic pipeline.

After the conversion from bcl to fastq format, the short reads were processed to clean up from sequencing adapters, and to filter or trim the reads for sequence quality (minimum Phred quality of 10 and minimum length of trimmed sequence of 30). Both these steps were performed with AdapterRemoval v.1.5.4 tool [35], after which the cleaned reads were mapped on human reference genome hg19 with Burrows–Wheeler Aligner (BWA v0.7.12) [36] for WES data, in paired-end or single-end mode according to the type of sample, and with the TopHat/Bowtie v2.0.9 [37] pipeline for RNA-seq data. Samtools v1.4 [38] was adopted to remove the optical and PCR duplicates and to index the alignment files. Then, the RNA-seq data analysis was performed to examine for the presence of any chromosomal rearrangements leading to gene fusions. For this purpose, four different tools were adopted: TopHat-Fusion v.2.0.9 [39], Defuse v0.6.2 [40], ChimeraScan v0.4.5 [41], FusionMap v2015 [42]. In order to increase the specificity, a predicted gene fusion was considered for further investigation if it was detected at least by two of four predictors. Gene expression profiling and differential expression of D842V mutant was evaluated comparing them with a set of seven GIST tumors with mutation on KIT exon 11 as previously described by Nannini et al. (2014) [43], and the pathway enrichment analysis was performed with WEB-based GEne SeT AnaLysis Toolkit (www.webgestalt.org) adopting the Reactome functional database (https://reactome.org/) as reference and the default settings of the tool. This analysis was run on the pre-ranked gene list of 1882 differentially expressed genes, selected by soft filtering cut-off (*p* < 0.05 and |log_2_Ratio| < 0.4). 

The whole exome data were then analyzed with the aim to detect point mutations and copy number variations. First, the realignment around insertions and deletions (InDels) and the base quality recalibration were performed with Genome Analysis Toolkit v3.3-0 (GATK) [44]. For WES tumor samples, Mutect v1.1.7 [45] and GATK v3.3-0 (HaplotypeCaller function) were then used to call the single nucleotide variants (SNVs) and InDels respectively, adopting the default parameters for both algorithms. Among the whole set of called variants, we selected those with total depth > 10, Ratio > 0.2 (ratio between the depth of coverage of the alternate event and the total depth of coverage), present within coding exons and having a non-silent effect on protein sequence (non-synonymous and nonsense SNVs, frameshift and non-frameshift InDels). The alterations were filtered on databases of human variability (dbSNP: https://www.ncbi.nlm.nih.gov/SNP/), 1000Genomes: http://www.internationalgenome.org/; EVS: http://evs.gs.washington.edu/EVS/; ExAC: http://exac.broadinstitute.org/) in order to discard polymorphism and keep novel or very rare variants (population frequency < 0.01). All the gene-based and filter-based annotation steps were performed with Annovar v2015Jun16 software tool [46] with the provided database. Finally, the resulting list of variants were distinguished between somatic or germline by calling the alternate events on the normal counterpart alignments using Samtools v1.4 mpileup function and assuming the following criteria: total depth > 5, depth of altered base = 0 (depth = 1 or depth = 2 were allowed if the total depth was ≥15 or ≥30 respectively) to include the variant in the “somatic” set. Otherwise we marked the events as “germline”, or “undetermined” if the total coverage on the normal counterpart was considered not sufficiently informative (total depth < 5). The somatic variants were handled with SnpSift dbNSFP v4.1 [47], an integrated database of functional computational tools to predict the alteration effects of on protein function and stability.

Moreover, on paired tumor/normal WES data, the analysis of amplifications and large deletions was performed applying two different tools, Control FREEC v7.2 [48] and ADTEX v2.0 [49]. A consensus method was implemented with the aim of selecting the overlapping regions given by the two algorithms (overlap of gain or loss ≥ 80%) followed by a downstream filtering procedure that takes into account the uncertainty value given by Control FREEC (uncertainty < 80%). Finally, the Database of human Genomic Variants (http://dgv.tcag.ca/dgv/app/home) was adopted to filter out the polymorphic copy number variants.

The 3D structure of PDGFRA D842V mutant was built with Modeller v9.8 [50] using the Protein Data Bank (PDB: https://www.rcsb.org/) crystal of tyrosine kinase domain of human PDGFRA (PDB: 5K5X). Chimera v1.11.2 [51] was adopted to visualize the model and to compare it with the wild-type structure by distance analyses (Structure Analysis—Distance function). The Imatinib–Kit human structure (PDB: 1T46) was also aligned to the PDGFRA mutant with the function MatchMaker.

The 3D model of the human PDGFRA in its active conformation was built by adopting the Kinase domain of KIT in the active conformation (PDB: 1PKG) as a template (sequence identity of 61%). After the generation of pairwise alignment using ClustalW2 (https://www.ebi.ac.uk/Tools/msa/clustalw2/) [52], 20 different models were computed with Modeller v9.8, and Discrete Optimized Protein Energy function was evaluated with the aim to select the lowest energy structure.

The Rigid Molecular Docking of crenolanib to the modeled PDGFRA was performed with Autodock v4.2 [53]. The ligand (Crenolanib) coordinates were generated with PRODRG server [54] and five active torsion angles were set. The 3D grid box (dimensions 60 × 60 × 60 unit in number of grid points; grid spacing 0.375 Å) centered into the kinase ATP binding pocket was then created using autogrid v4.2. A total of 1500 runs of Lamarckian genetic algorithm were performed, with an initial population of 300 conformations, a cutoff of 27,000 generations, and with rates of mutation and crossover set to 0.02 and 0.8, respectively. The final solution was characterized by the lowest binding energy.

## Figures and Tables

**Table 1:** Patients and Tumor Samples.

Patient	Sample	Age at Surgery	Sex	Type of Sample	Primary Tumor Location	Tumor Size	Mitotic Index 50HPF	Source	Treatment Before Sampling	WES	RNA-Seq
P01	T01	62	M	Frozen	stomach	11	300	metastasis	C, D	101X1	NA
P02	T02	74	F	Frozen	stomach	16.2	5	metastasis	C	101X1	NA
P03	T03	51	M	Frozen	stomach	5	55	metastasis	I, G/D, Su, IPI-504, So, STA-9090, D, GDC-0980, C	101X1	NA
P04	T04	51	M	FFPE	stomach	6.2	7	metastasis	none	101X1	NA
	T05	51	M	FFPE	stomach	6.2	7	metastasis	none	101X1	NA
P05	T06	53	M	Frozen OCT	NA	NA	NA	primary	none	101X1	NA
P06	T07	56	M	FFPE	stomach	30	high	metastasis	I, Su, So, N	101X1	NA
	T08	56	M	FFPE	stomach	30	high	metastasis	I, Su, So, N	101X1	NA
	T09	56	M	FFPE	stomach	30	high	metastasis	I, Su, So, N	101X1	NA
	T10	56	M	FFPE	stomach	30	high	metastasis	I, Su, So, N	101X1	NA
	T11	56	M	FFPE	stomach	30	high	metastasis	I, Su, So, N	101X1	NA
P07	T12	63	F	Frozen	stomach	10.5	19	NA	C	101X1	NA
P08	T13	76	M	Frozen	stomach	NA	NA	primary	none	101X1	NA
P09	T14	30	M	Frozen	stomach	NA	NA	primary	none	101X1	NA
P10	T15	50	F	Frozen	stomach	1.8	2	primary	none	100X2	75X2
P11	T16	75	F	Frozen	stomach	7	8	primary	none	100X2	75X2
P12	T17	68	M	Frozen	stomach	3	5	primary	none	100X2	75X2
P13	T18	76	M	Frozen	stomach	4.5	6	primary	none	100X2	75X2
P14	T19	51	M	Frozen	stomach	12	2	primary	none	100X2	75X2

WES, whole exome sequencing; FFPE, formalin-fixed paraffin-embedded; OCT, optimal cutting temperature; M, male; F, female (F); NA, Not Available; C, Crenolanib; D, dasatinib; I, imatinib; N, nilotinib; So, sorafenib; Su, sunitinib; GD, gemcitabine/docetaxel ; STA-9090, ganetespib; GDC-0980, apitolisib; IPI-504, retaspimycin.

**Table 2:** Reactome gene set enrichment performed with WebGestalt.

Pathway	Reactome Gene Set	Description	# Genes (Total)	# Leading-Edge Genes	NES	*p* Value	FDR
Positively related Pathways	R-HSA-373076	Class A/1 (Rhodopsin-like receptors)	30	24	2.03	<0.001	0.0058
	R-HSA-375276	Peptide ligand-binding receptors	18	14	1.94	0.001	0.014
	R-HSA-500792	GPCR ligand binding	38	27	1.90	<0.001	0.01975
	R-HSA-1630316	Glycosaminoglycan metabolism	19	13	1.77	0.001	0.09927
	R-HSA-388396	GPCR downstream signaling	70	35	1.74	0.001	0.13397
	R-HSA-3560782	Diseases associated with glycosaminoglycan metabolism	6	6	1.70	0.005	0.15244
	R-HSA-1638091	Heparan sulfate/heparin (HS-GAG) metabolism	9	8	1.71	0.003	0.15294
	R-HSA-372790	Signaling by GPCR	99	45	1.68	<0.001	0.16662
	R-HSA-1296072	Voltage gated Potassium channels	6	4	1.66	0.003	0.19534
	R-HSA-112314	Neurotransmitter Receptor Binding And Downstream Transmission In The Postsynaptic Cell	17	10	1.65	0.014	0.19979
							
Negatively related Pathways	R-HSA-1640170	Cell Cycle	56	49	−2.83	<0.001	<0.001
	R-HSA-69242	S Phase	15	15	−2.53	<0.001	0.00088
	R-HSA-69278	Cell Cycle, Mitotic	41	37	−2.57	<0.001	0.00131
	R-HSA-2559583	Cellular Senescence	13	13	−2.46	<0.001	0.00162
	R-HSA-69239	Synthesis of DNA	14	14	−2.47	<0.001	0.00202
	R-HSA-69306	DNA Replication	14	14	−2.42	<0.001	0.00266
	R-HSA-69481	G2/M Checkpoints	18	17	−2.29	<0.001	0.00723
	R-HSA-73894	DNA Repair	29	28	−2.22	<0.001	0.00920
	R-HSA-5693538	Homology Directed Repair	16	15	−2.21	<0.001	0.01075
	R-HSA-5693532	DNA Double-Strand Break Repair	16	15	−2.18	<0.001	0.01076

In the red box are listed the top 10 positively related (enriched) pathways in D842V mutant Gastrointestinal Stromal Tumor (GIST), while the negatively related (depleted) pathways are listed in the green box. GPCR, G-protein-coupled receptor; NES, normalized enrichment score; FDR, false discovery rate.

## Supplementary Materials

Supplementary materials can be found at http://www.mdpi.com/1422-0067/19/3/732/s1.
